# What the papers say

**DOI:** 10.1093/jhps/hnz016

**Published:** 2019-05-06

**Authors:** Ajay Malviya

**Affiliations:** Consultant Orthopaedic Surgeon, Northumbria Healthcare NHS Foundation Trust, Senior Lecturer, Regenerative Medicine—ICM, Newcastle University, 10 East Brunton Wynd, Newcastle upon Tyne, UK

The *Journal of Hip Preservation Surgery* (*JHPS*) is not the only place where work in the field of hip preservation may be published. Although our aim is to offer the best of the best, we continue to be fascinated by work that finds its way into journals other than our own. There is much to learn from it so *JHPS* has selected six recent and topical subjects for those who seek a summary of what is taking place in our ever-fascinating world of hip preservation. What you see here are the mildly edited abstracts of the original articles, to give them what *JHPS* hopes is a more readable feel. If you are pushed for time, what follows should take you no more than 10 min to read. So here goes…

## CAN HIGH-GRADE CARTILAGE DAMAGE BE PREDICTED IN THE CLINIC?

The preoperative assessment of cartilage lesions is critical to surgical planning and decision-making. The accurate radiographic determination of acetabular cartilage damage has remained elusive for modern imaging modalities, including magnetic resonance imaging (MRI) and magnetic resonance arthrography (MRA). While risk factors have been individually described, no multivariable system exists for predicting high-grade cartilage damage. Researchers from the Mayo clinic [1] aimed to determine the preoperative predictors of grade 3–4 acetabular labrum articular disruption (ALAD) lesions.

In this case–control study, radiographs were reviewed from primary hip arthroscopic procedures performed at two high-volume institutions between December 2007 and April 2017. The predictive value of demographic and radiographic factors for the intraoperative documentation of ALAD grade 3–4 damage was analysed and entered into a multivariable model, and a statistically guided scoring system for the damage risk was created using the Akaike information criterion. The scoring system was then prospectively validated on 167 patients who underwent primary hip arthroscopy between April 2017 and February 2018.

A total of 652 primary hip arthroscopic procedures in 614 patients (390 females, 224 males; mean age 33.2 years; mean body mass index 26.9 kg m^−^^2^) from 2007 to 2017 were analysed. Male sex (odds ratio [OR] 3.11; *P* <0.01), age ≥35 years (OR 1.96; *P* <0.01), cam morphology (alpha angle >55°) (OR 2.96; *P* <0.01) and Tönnis grade 1–2 (grade 1: OR 4.14; *P* <0.01 and grade 2: OR 9.29; *P* <0.01) were univariate risk factors for intraoperatively documented high-grade damage. A multivariable scoring system, the rapidly assessed predictor of intraoperative damage (RAPID) score (0–5 points), was generated based on sex, Tönnis grade and cam morphology. Patients with increasing RAPID scores had an increasing risk of damage, with a 10.5% risk for those with 0 points and an 88.0% risk for those with five points (*P* <0.01). The area under the curve was 0.75 for the study group and 0.76 for the validation group (*P* =0.94).

The authors concluded that while preoperative MRI has diagnostic value for hip arthroscopic surgery, the RAPID score provides added benefit as a readily employable, in-clinic system for predicting high-grade cartilage damage. The discriminatory value of the RAPID score compares favourably with previous MRI and MRA studies. This information will help the clinician and patient plan for high-grade damage and identify potential targets for cartilage treatment.

## CAN WE USE BIOMARKERS TO ASSESS THE EFFICACY OF SURGERY FOR FEMOROACETABULAR IMPINGEMENT?

The early recognition and management of patients with hip lesions, such as femoroacetabular impingement (FAI) and early hip osteoarthritis (OA), may preempt significant hip morbidity. The identification of reliable biomarkers may help guide decision making in an efficient and cost-effective manner.

In a multi-centred work from United States, the authors [2] set out to determine the biomarkers that have been associated with FAI as well as identify serum, synovial and urinary analytes that have shown clinical utility in the prediction or identification of hip OA. In this systematic review and meta-analysis, the authors identified, seven articles that could be included. Pooled estimates were calculated utilizing a fixed-effects inverse-variance model weighted for individual study size.

A total of 1747 patients with a mean age of 37.5 ± 4.5 years (76.4% female) were identified. Forty-three unique biomarkers were assessed. Although general proinflammatory cytokines IL-1 and TNF-α exhibited inconsistent trends in arthritic hips, IL-6 demonstrated a consistent increase (+84.8%; *P* <0.05). A significant difference was found in levels of the fibronectin–aggrecan complex (FAC) in patients with OA compared with controls (0.08 ± 0.40 versus 1.15 ± 0.35 μg ml^−1^, respectively; *P* <0.001). It was the only specific analyte to show a significant difference between those with and without OA. In the setting of FAI, cartilage oligomeric matrix protein (COMP) was significantly increased in athletes after adjusting for concurrent knee and hip OA. A statistically significant difference (*P* <0.05) was present in FAI-positive hips (9.0 ± 0.1) compared with controls (8.4 ± 0.1). Other biomarkers, such as CXCL3, which exhibited statistically significant differences compared with controls, did not control for underlying factors such as age and concomitant lesions.

The study concluded that COMP and FAC are specific biomarkers with potential utility in the diagnosis and management of FAI and hip OA, given their ability to differentiate between controls and patients with hip lesions. Further research is necessary to identify their ability in determining disease severity, predicting the response to treatment and establishing an association with the risk of long-term OA.

## ACETABULAR CHONDRAL LESIONS ASSOCIATED WITH FAI TREATED BY AUTOLOGOUS MATRIX-INDUCED CHONDROGENESIS OR MICROFRACTURE: A COMPARATIVE STUDY AT 8-YEAR FOLLOW-UP

The aim of this retrospective study from Italy [3] was to investigate, at 8 years, the clinical follow-up and failure rate (revision rate/conversion to arthroplasty) of patients with hip chondral lesions associated with FAI and to compare over time the treatment by microfracture (MFx) and autologous matrix-induced chondrogenesis (AMIC).

Patients aged between 18 and 55 years, with acetabular grade III and IV chondral lesions (Outerbridge), measuring 2–8 cm^2^ operated on at least 8 years before enrolment. Exclusion criteria were rheumatoid arthritis, dysplasia or axial deviation of the femoral head. There were no arthritic lesions, Tonnis <2 or joint space of at least 2 mm. MFx was performed with an awl, and the Chondro-Gide membrane used for the AMIC procedure was placed without glue. Outcomes used modified Harris hip score (mHHS) at 6 months and yearly for 8 years and patient acceptable symptomatic state.

Among 130 patients, 109 fulfilled inclusion criteria. Fifty were treated by MFx and 59 by AMIC. The mHHS significantly improved in both groups from 46 to 78 for mHHS at 6–12 months, even for lesions >4 cm^2^. From 2 to 8 years, mHHS in the AMIC group was better than in the MFx group (*P* <0.005). This mHHS improvement in the AMIC group was maintained through the 8-year follow-up period, whereas it deteriorated after 1 year in the MFx group (*P* <0.005). Eleven patients (22%) in the MFx group required total hip arthroplasty (THA); none in the AMIC group did. Patient acceptable symptomatic state analysis confirmed similar short-term improvement, but a significant (*P* <0.007) degradation after 2–8 years in MFx patients.

The authors concluded that both MFx and AMIC techniques led to marked clinical short-term improvement in patients with chondral defects resulting from FAI in the first 2 years. However, AMIC gave significantly better results as measured by mHHS, which were maintained after 8 years, the results of MFx in the hip deteriorated over time with 22% of patients undergoing conversion to THA. No patient in the AMIC group was converted to THA; the results of AMIC appeared stable over time and independent of lesion size.

## TEN- AND 20-YEAR SURVIVORSHIP OF THE HIP AFTER PERIACETABULAR OSTEOTOMY FOR ACETABULAR DYSPLASIA

This cross-sectional retrospective study has looked at the long-term outcome of the Bernese periacetabular osteotomy (PAO) for acetabular dysplasia in terms of survival, functional outcomes and radiographic parameters.

The study from UCLA, United States [4] includes 430 patients who were operated between 1987 and 2014 but data were available for 302 hips in 258 patients from medical records and/or mail-in/phone questionnaires. Functional outcome data consisted of postoperative Hip Osteoarthritis Outcome Score and University of California-Los Angeles Activity Score. Pre- and postoperative radiographs were used to determine lateral center-edge angle, anterior center-edge angle, Tönnis angle/grade and head-to-ilioischial line distance. Survivorship of the native hip was determined by Kaplan–Meier analysis.

Of the 302 hips analysed, 248 were still surviving native hips and 54 had gone on to a THA at the time of data acquisition. The average age of patients in the entire cohort at PAO was 32.7 years (range 13–63 years). Of the 258 patients, 215 were female patients (83.3%) and 43 male patients (16.8%). The average age of patients in the surviving group at PAO was 32.3 years, and the average age of patients in the THA group was 36.6 years (*P* < 0.01). At the time of data acquisition, follow-up ranged from 2 to 27 years (average 11.2 years). Radiographic analyses for surviving and failed hips revealed that pre- and postoperative Tönnis grade was statistically significant predictors for conversion to THA (*P* < 0.01). Survivorship of the native hip was 86% at 10 years and 60% at 20 years in the surviving cohort. Survivorship stratified by age at the time of PAO demonstrated a 10-year survivorship of 93.3, 90.1, 81.6 and 63.2% at ages 20, 30, 40 and 50 years, respectively. No notable difference exists in survivorship between male and female patients; however, male patients had a trend toward lower survivorship compared with female patients at 15 years.

The authors concluded that in their cohort of 302 hips, older age at the time of PAO and higher Tönnis grade were negative prognostic factors for joint survival after PAO. Surviving hips after PAO have good functional outcomes even up to 20 years after surgery. This survivorship analysis represents one of the largest and longest survival studies of patients after PAO outside of the results reported from Bern.

## DOES EXTRACORPOREAL SHOCK WAVE THERAPY (ESWT) HELP IN TREATMENT OF GREATER TROCHANTERIC PAIN SYNDROME (GTPS)

In a randomized controlled trial, investigators from the University of Pavia, Italy [5] aimed to explore the role of focused ESWT in the treatment of GTPS. A total of 50 patients affected by GTPS with gluteal tendinopathy were recruited; the study group was assigned to receive f-ESWT, the control group received ultrasound therapy (UST). Hip pain and lower limb function was assessed by means of a numeric rating scale (p-NRS) and the Lower Extremity Functional Scale (LEFS scale), respectively. The first follow-up evaluation (2M-FUP) was performed 2 months after the first treatment session, the second (6M-FUP) was carried out 6 months later.

The mean age of the population was 61.24 (9.26) years. A marked prevalence of the female sex was recorded (44 subjects, 86%). The statistical analysis showed a significant pain reduction over time for the study group and the control group, the f-ESWT proving to be significantly more effective than UST (*P* < 0.05) at the 2M-FUP (2.08 versus 3.36) and at the 6M-FUP (0.79 versus 2.03). A marked improvement of the LEFS total score was observed in both groups as well, without any statistically significant difference.

The authors concluded that f-ESWT is effective in reducing pain, both in the short-term and in the mid-term perspective and also observed a functional improvement in the affected lower limb, but, in this case, f-ESWT showed not to be superior to UST.

## DOES PELVIC TILT INFLUENCE GROIN INJURY IN ATHLETES??

The role of spino-pelvic alignment in patients with hip pathology is topical, but it is not clear if it will have an influence in athletes presenting with groin injury. Van Goeverden *et al*. [6] from the Netherland, performed a case–control study to determine whether athletes with groin injury had less active pelvic tilt (APT) than non-injured controls. The study includes 17 athletes (Tegner > 5, mean age 25.1 years) with groin injury and 27 healthy controls (Tenger > 5, mean age 24.4 years). The main outcome measure was active pelvic tilt, defining the ability of an individual to actively tilt the pelvis anteriorly and posteriorly over a frontal axis, and hip range of motion (HROM) parameters.

Linear regression model associations with generalized estimated equations revealed that APT was lower on injured sides compared with non-injured for total (21.1 versus 27.2, *P*=0.003) and anterior (10.2 versus 13.7, *P*=0.004) APT. Posterior APT (‒10.9 versus ‒13.4, *P*=0.06) showed a trend towards being lower in those with groin injury. HROM parameters were not found associated.

The authors concluded that the total active and anterior pelvic tilt were lower on the injured side in athletes with groin injury when compared with non-injured sides and healthy controls. This may be a relevant factor to consider in rehabilitation. However, whether this is a cause or effect cannot be ascertained due to the cross-sectional study design, and perhaps a further prospective study on a larger cohort of athletes is on the cards.

## CONFLICT OF INTEREST STATEMENT

None declared.
